# How do stone attenuation and skin-to-stone distance in computed tomography influence the performance of shock wave lithotripsy in ureteral stone disease?

**DOI:** 10.1186/s12894-015-0069-7

**Published:** 2015-07-23

**Authors:** Gautier Müllhaupt, Daniel S. Engeler, Hans-Peter Schmid, Dominik Abt

**Affiliations:** Department of Urology, Cantonal Hospital St. Gallen, Rorschacherstrasse 95, 9007 St. Gallen, Switzerland

**Keywords:** Ureteral stones, Treatment outcome, Shock wave lithotripsy, Hounsfield Units, Skin-to-stone distance

## Abstract

**Background:**

Shock wave lithotripsy (SWL) is a noninvasive, safe, and efficient treatment option for ureteral stones. Depending on stone location and size, the overall stone-free rate (SFR) varies significantly. Failure of stone disintegration results in unnecessary exposure to shock waves and radiation and requires alternative treatment procedures, which increases medical costs. It is therefore important to identify predictors of treatment success or failure in patients who are potential candidates for SWL before treatment. Nowadays, noncontrast computed tomography (NCCT) provides reliable information on stone location, size, number, and total stone burden. The impact of additional information provided by NCCT, such as skin-to-stone distance (SSD) and mean attenuation value (MAV), on stone fragmentation in ureteral stone disease has hardly been investigated separately so far. Thus, the objective of this study was to assess the influence of stone attenuation, SSD and body mass index (BMI) on the outcome of SWL in ureteral stones.

**Methods:**

We reviewed the medical records of 104 patients (80 men, 24 women) with ureteral stone disease treated consecutively at our institution with SWL between 2010 and 2013. MAV in Hounsfield Units (HU) and SSD were determined by analyzing noncontrast computed tomography images. Outcome of SWL was defined as successful (visible stone fragmentation on kidney, ureter, and bladder film (KUB)) or failed (absent fragmentation on KUB).

**Results:**

Overall success of SWL was 50 % (52 patients). Median stone attenuation was 956.9 HU (range 495–1210.8) in the group with successful disintegration and 944.6 (range 237–1302) in the patients who had absent or insufficient fragmentation. Median SSD was 125 mm (range 81–165 mm) in the group treated successfully and 141 mm (range 108–172 mm) in the patients with treatment failure. Unlike MAV (*p* = 0.37), SSD (*p* < 0.001) and BMI (*p* = 0.008) significantly correlated with treatment outcome.

**Conclusion:**

The choice of treatment for ureteral stones should be based on stone location and size as considered in the AUA and EAU guidelines on urinary stone disease. In ambiguous cases, SSD and BMI can be used to assist in the decision. In this study, MAV showed no correlation with fragmentation rate of SWL.

## Background

Shock wave lithotripsy (SWL) is a noninvasive, safe, and efficient treatment option for ureteral stones. Depending on stone location and size, the overall stone-free rate (SFR) varies significantly, leading to corresponding recommendations in the guidelines of the American Urological Association and the European Association of Urology: For proximal ureteral stones <10 mm, SWL has a higher SFR than ureterorenoscopy (URS), while URS seems to be superior for stones >10 mm. For mid-ureteral stones, URS appears to be superior, with statistical limitations, because fewer patients have been investigated. For distal stones >10 mm, URS is the treatment of choice, while SWL and URS are options for small stones [1, 2]. Failure of stone disintegration results in unnecessary exposure to shock waves and radiation, further patient suffering, and requires alternative treatment procedures, which increases medical costs [3]. It is therefore important to identify predictors of treatment success or failure in patients who are potential candidates for SWL before treatment.

Radiographic assessment of the stone is required to decide on the best treatment. Nowadays, noncontrast computed tomography (NCCT) provides reliable information on stone location, size, number, and total stone burden, and is therefore recommended as the standard diagnostic tool in urinary stone disease [3, 4]. Moreover, several studies have shown an impact of mean attenuation value (MAV) on treatment success of SWL in kidney stones, leading to corresponding guideline recommendations [1, 2]. Despite the widespread use of NCCT, however, the impact of additional information provided by NCCT, such as skin-to-stone distance (SSD) and MAV, on stone fragmentation in ureteral stone disease has hardly been investigated separately so far [5–8]. Moreover, as limiting factors, three of the four studies reported on so far covered only one SWL session regardless of whether disintegration occurred or not, and treatment success was analyzed in all four studies at the earliest 2 weeks after SWL. The study by Ng et al. also included only proximal ureteral stones, and no real-time fluoroscopic screening was performed during treatment [7].

Thus, the objective of this study was to determine how additional information provided by NCCT and patient’s physical constitution might influence fragmentation rate of SWL in ureteral stone disease.

Table 1 shows a summary of the literature.

**Table 1:** Review of the literature

				Prediction of successful disintegration/treatment			
References	Year	Stone location	n All/Renal/Ureteral	Mean attenuation value (MAV) All/Renal/Ureter	SSD All/Renal/Ureter	BMI All/Renal/Ureter	Cut off MAV/SSD/BMI
Joseph et al. [14]	2002	Renal	30/30/-	Yes/Yes/-	-/-/-	No/No/-	Renal: 950 HU/-/-
Pareek et al. [6]	2003	Renal and ureteral	50/20/30	Yes/Yes/Yes	-/-/-	No/-/-	Ureteral: 900 HU/-/-
Wang et al. [15]	2005	Renal	80/80/-	Yes/Yes/-	-/-/-	-/-/-	Renal: 900HU/-/-
Gupta et al. [16]	2005	Renal and proximal ureter	108/89/19	Yes/-/-	-/-/-	-/-/-	All: 750 HU/-/-
Yoshida et al. [17]	2006	Renal and proximal ureter	56/25/31	Yes/-/-	-/-/-	-/-/-	-/-/-
El Nahas et al. [3]	2007	Renal	120/120/-	Yes/Yes/-	Yes/Yes/-	Yes/Yes/-	Renal: 1000HU/-/-
Perks et al. [11]	2008	Renal	111/111/-	Yes/Yes/-	Yes/Yes/-	No/No/-	Renal: 900HU/9 cm/-
Ng et al. [7]	2009	Proximal ureter	94/-/94	Yes/-/Yes	Yes/-/Yes	No/-/No	Renal:593 HU/9.2 cm/-
Patel et al. [10]	2009	Renal	83/83/-	No/No/-	Yes/Yes/-	-/-/-	Renal: −/10 cm/-
Wiesenthal et al. [5]	2010	Renal and ureteral	422/218/204	Yes/Yes/Yes	Yes/Yes/Yes	Yes/No/Yes	All: 900 HU/ 11 cm/-
Park et al. [12]	2010	Renal	115/115/-	Yes/Yes/-	No/-/-	-/-/-	Renal: 863 HU/-/-
Shah et al. [18]	2010	Renal and proximal ureter	99/71/28	Yes/-/-	-/-/-	-/-/-	-/-/-
Tanaka et al. [13]	2013	Renal and ureteral	75/27/48	Yes/-/-	No/-/-	No/-/-	All: 780 HU/-/-
Celik et al. [8]	2015	Renal and ureteral	254/123/131	Yes/Yes/Yes	-/Yes/	-/Yes/-	Renal: 750 HU/-/-
Nakasato et al. [19]	2015	Renal and ureteral	260/92/168	Yes/-/-	No/-/-	-/-/-	All: 815 HU/-/-

## Methods

**Fig. 1:**
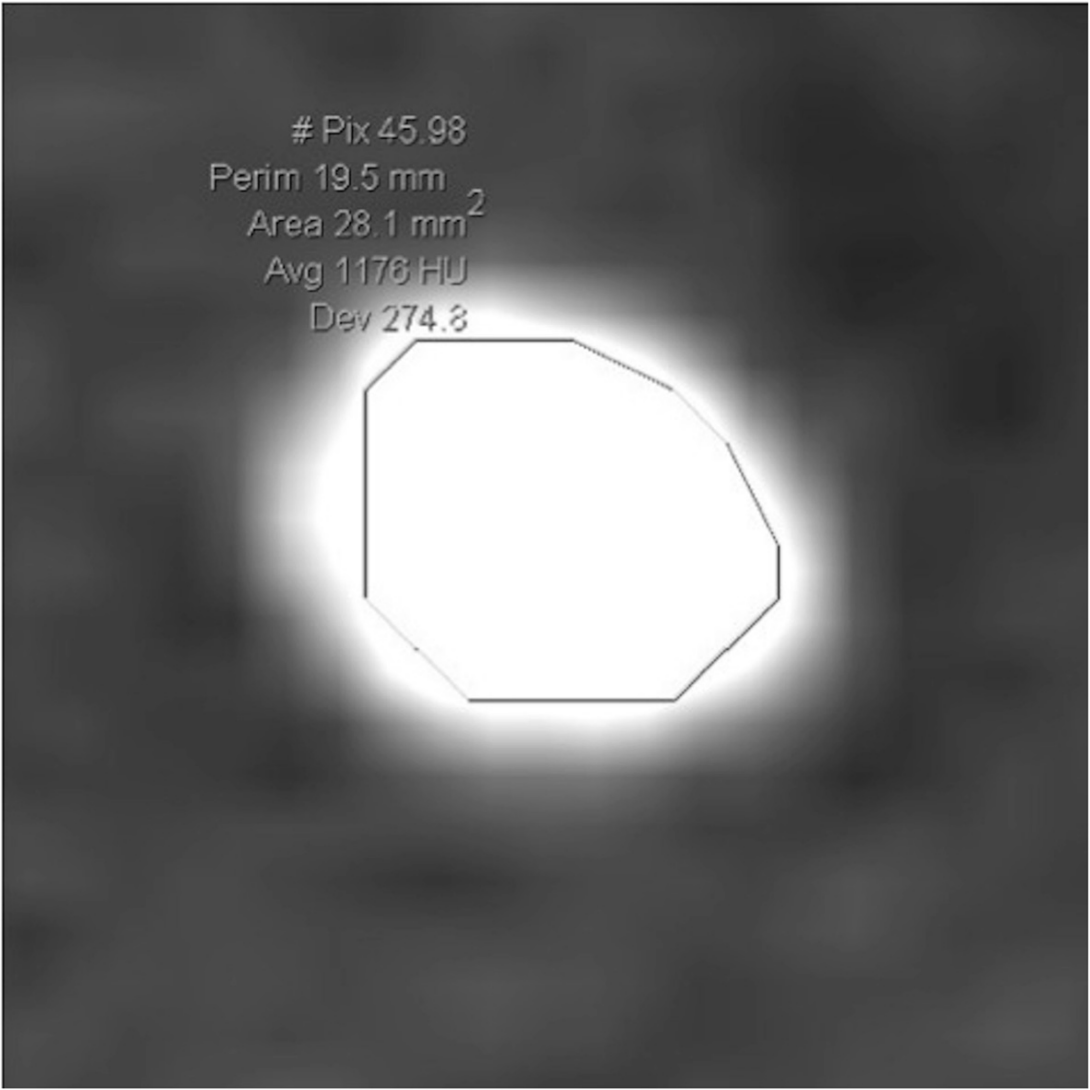
Defined regions of interest just smaller than the stone without including adjacent soft tissue

**Fig. 2:**
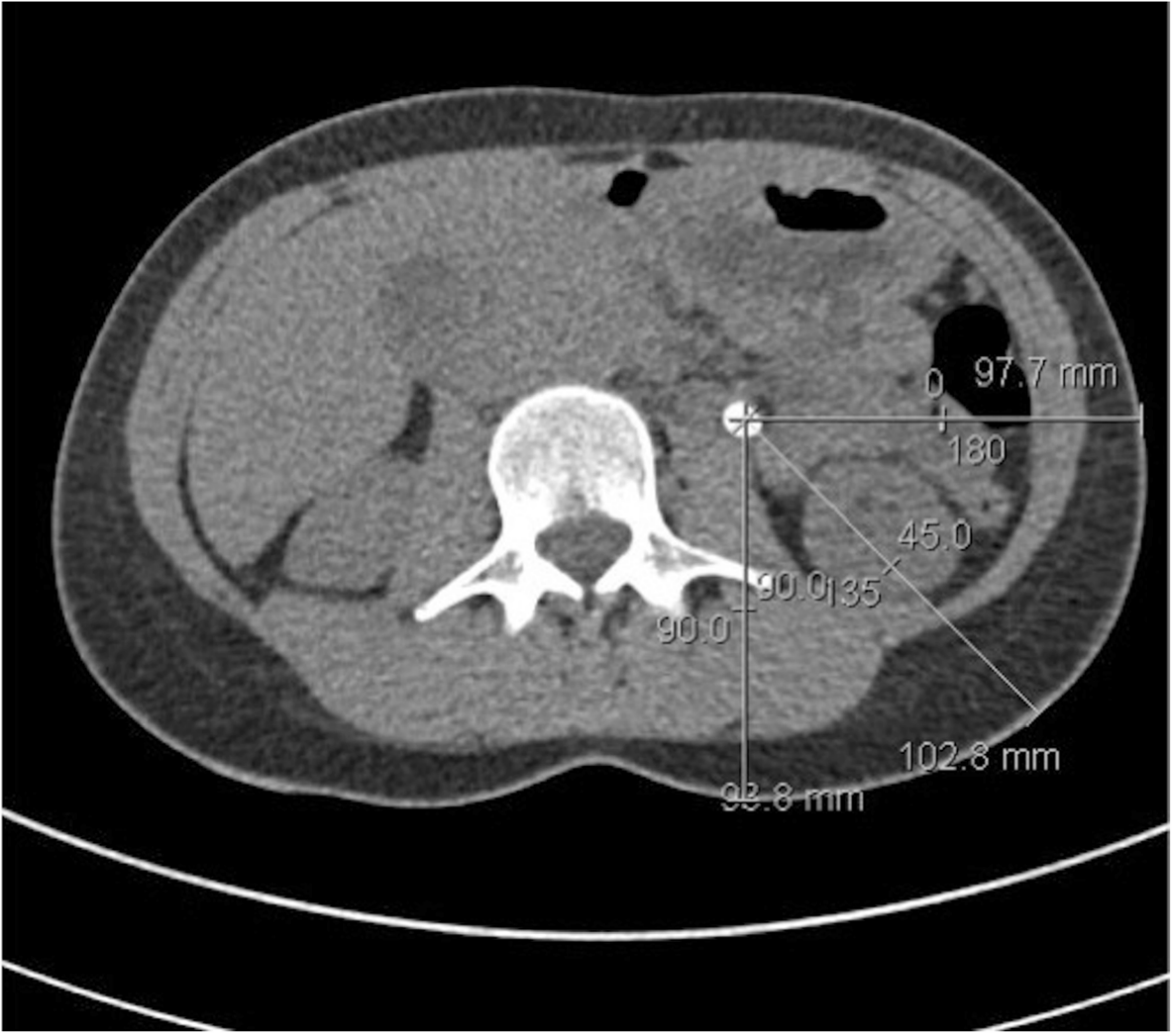
Skin-to-stone distance (SSD) was calculated by the measuring distances from Stone-to-skin at 0°, 45°, and 90° using radiographic calipers

One hundred four patients treated consecutively with SWL for distal and proximal ureteral stones in our department between January 2010 and December 2013 were included in this retrospective study. Data analysis was conducted according to the declaration of Helsinki and approved by the Local Ethics Committee of St. Gallen (EKSG 15/055). Written informed consent for data analysis was obtained. NCCT was performed before treatment using a multidetector row helical CT scanner (Siemens, Definition Flash, Forchheim, Germany) with 30–460 mA, 120 kV and 2 mm collimation in every patient. As suggested in a study by Eisner et al. [9], stone size and Hounsfield Unit (HU) measurements were obtained in a standard bone window (window width-1,120 and window level-300). The image with the largest stone diameter was used to define maximum stone size. MAV was obtained by measuring the mean HU of defined regions of interest just smaller than the stone in magnified images without including adjacent soft tissue on each slice of the axial planes (Fig. 1). SSD was calculated as described by El Nahas et al. [3] and the distances at 0°, 45° and 90° were measured using radiographic calipers (Fig. 2). The average was calculated as the SSD. The measurements were performed analogous in prone position when targeting pelvic stones. The SSD was also measured and evaluated at an angle of 90° separately, as this seems to be the most important angle in the setting of the SLX-F2 (Storz Medical, Tägerwilen, Switzerland) which was used to perform SWL under sedoanalgesia.

If tolerated by the patient, up to 4,000 shocks (60–90/min) with an energy level of up to 8 according to the manufacturer’s scale were delivered during each SWL session. The energy level 8 corresponded to 16.4 kV with the precise focus and 12.8 kV with the extended focus. In patients with pain resistant to analgesic treatment, the energy and number of shocks were reduced according to the patient’s tolerance. Stones were targeted and fragmentation was monitored by biplanar fluoroscopy at regular intervals during treatment.

Patients were further evaluated by kidney, ureter, and bladder (KUB) film, renal ultrasound, and sieving of urine to assess fragmentation, the presence of renal dilatation and expulsion of ureteral stones the day after the respective session. In cases of missing or inadequate disintegration in KUB, SWL was repeated once or twice at intervals of 1 day. The clinical outcome was defined as successful (visible stone fragmentation on KUB) or failed (absent fragmentation on KUB) immediately after the last SWL session.

The correlation with and influence of a range of baseline characteristics on treatment outcome of SWL was examined: patient’s age, gender, weight, and BMI; stone location and volume, MAV, SSD; use of alpha blockers; presence of ureteral stents. Both univariate (chi-square or Mann–Whitney U-tests for dichotomous or continuous variables) and multivariate (binary logistic regression) analyses were performed to define significant factors. ROC curves were used for the determination of the best cut-off values. All tests were two-sided and a p-value of <0.05 was necessary to reject the null-hypothesis. Statistical analyses were performed using IBM SPSS Statistics Version 22 (IBM Corp., New York, U.S.A.).

## Results

A total of 104 consecutive patients were included (24 women, 80 men), median age was 45.5 years (range 19–80 years). Median largest diameter of stones was 6 mm (range 2–15 mm) and median MAV 949.3 HU (range 237–1302 HU).

The stones were located in the proximal ureter in 73 (70.2 %) patients and the distal ureter in 31 (29.8 %). Median BMI was 26.2 (range 17.4–37.0), median SSD (0°/45°/90°) 131.5 mm (range 81–172 mm) and median SSD (90°) 119 mm (range 76–161 mm).

Stone fragmentation was visible in 52 (50 %) patients and was not visible in the remaining patients, of whom 49 (94.2 %) needed further treatment: 43 (82.7 %) by URS, 4 (7.7 %) by ureteral stent insertion, and 2 (3.8 %) by further cycles of SWL. The three patients who needed no further treatment showed spontaneous stone passage during the treatment with SWL without stone disintegration.

Of the 52 patients who showed good stone fragmentation, 13 (25 %) needed further treatment by URS (8 patients, 15.4 %,), ureteral stent insertion (1 patient, 1.9 %) or further cycles of SWL (4 patients, 7.7 %) because of impacted fragments or distal steinstrasse.

Median MAV was 956.9 HU (range 495–1210.8 HU) in patients with good stone fragmentation and was 944.6 HU (range 237–1302 HU) in patients showing no stone fragmentation and requiring further treatment. In univariate analysis, MAV showed no correlation with stone fragmentation (*p* = 0.373).

Median BMI, SSD (0°/45°/90°) and SSD (90°) in patients with good stone fragmentation were 25.5 (range 17.4–35.0), 125 mm (range 81–165 mm) and 114.5 mm (range 76–159 mm). In patients without stone fragmentation, median BMI, SSD (0°/45°/90°) and SSD (90°) were 27.1 (range 21.1–37.0), 141 mm (range 108–172 mm) and 130 (range 85–161). In univariate analysis, BMI (*p* = 0.008), SSD (0°/45°/90°) (*p* < 0.001) and SSD (90°) (*p* < 0.001) significantly correlated with stone fragmentation.

In addition, maximum energy delivered showed a significant correlation with disintegration outcome (*p* = 0.015). Median energy level was 6 (range 4–8) in patients with good stone fragmentation and 6.4 (range 5–8) in patients with no stone fragmentation.

The results of univariate analyses are summarized in Table 2.

**Table 2:** Results of univariate analysis

Characteristic	Successful disintegration	Unsuccessful disintegration	*p*-value
Number of patients (%)	52 (50 %)	52 (50 %)	-
Age, years (median, range)	43.5 (19–80)	47.5 (22–77)	0.136
Gender, M/F (N/%)	35 (67.3 %)/17 (32.7 %)	45 (86.5 %)/7 (13.5 %)	0.035
Weight, kg (median, range)	73 (49–116)	85 (58–120)	<0.001
BMI, kg/m^2^ (median, range)	25.5 (17.4–35.0)	27.1 (21.6–37.0)	0.008
Skin-to-stone distance, mm, mean of 0°, 45° and 90° (median, range)	125 (81–165)	141 (108–172)	<0.001
Skin-to-stone distance, mm, 90° (median, range)	114.5 (76–159)	130 (85–161)	<0.001
Mean attenuation value, HU (median, range)	956.9 (495–1210.8)	944.6 (237–1302)	0.373
Stone size, mm (median, range)	7 (3–15)	6 (2–12)	0.071
Location, proximal/distal (N, %)	36 (69.2 %)/16 (30.8 %)	37 (71.2 %)/15 (28.8 %)	1.000
SWL cycles (median, range)	2 (1–3)	2 (1–3)	0.786
Number of shockwaves (median, range)	8000 (1000–12000)	8000 (3000–14000)	0.583
Power/Intensity Level (median, range)	6 (4–8)	6.4 (5–8)	0.015
Ureteral stent in place (N, %)	15 (28.8 %)	13 (25 %)	0.825
Alpha-blocker (N, %)	42 (80.8 %)	38 (73.1 %)	0.486
Secondary procedures			
URS (N, %)	8 (15.4 %)	43 (82.7 %)	-
Ureteral stent (N, %)	1 (1.9 %)	4 (7.7 %)	-
SWL (N, %)	4 (7.7 %)	2 (3.8 %)	-

According to multivariate analyses, SSD (90°) was a significant predictor for disintegration failure (regression coefficient: −0.046, standard error: 0.013, odds ratio 0.955, 95 % confidence interval: 0.930–0.980, p-value < 0.001). Moreover, maximum delivered energy tended to be lower in patients with successful disintegration than in patients with disintegration failure without reaching statistical significance (regression coefficient: −0.528, standard error: 0.272, odds ratio 0.590, 95 % confidence interval: 0.346–1.004, p-value < 0.052). Weight and BMI of the patient were not included in the multivariate analysis because of multicollinearity with SSD.

**Fig. 3:**
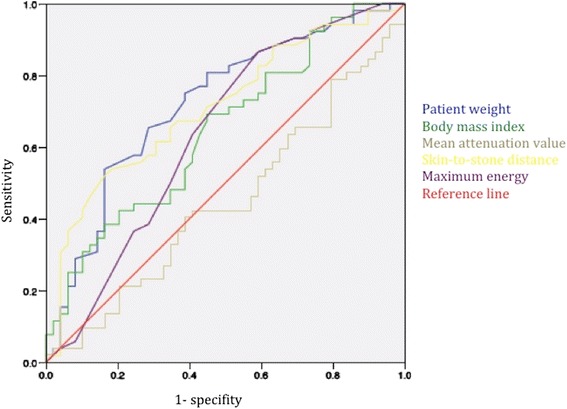
ROC curves

The ROC curves for different parameters were analyzed to find the optimum cut-off values to predict disintegration failure (Fig. 3). The optimum cut-off point for SSD (90°) would be >11.9 cm (sensitivity 65.4 %, specificity 65.3 %), for patient weight >82.5 kg (sensitivity 65.4 %, specificity 71.4 %), and for BMI >25.9 kg/m^2^ (sensitivity 69.2 %, specifity 55.1 %).

## Discussion

The results of this study show that SSD and BMI are significant predictors of the outcome of SWL. As described earlier by Patel et al. for kidney stones [10], we found no significant association between MAV and fragmentation rate of SWL for ureteral stones.

The use of NCCT for diagnosis of ureteral stones is well established and a common practice worldwide [7]. The method of measuring SSD in NCCT has been well described in the literature and there are only marginal differences between studies with regard to the method [3, 5–7, 10–13].

The method for determining MAV has been described inconsistently, however. For example, Joseph et al. [14] used a calculous pixel map of 100 attenuation values in a 10 x 10 matrix in unenhanced axial NCCT section, while Wiesenthal et al. [5] measured attenuation values using bone windows on the magnified, axial image of the stone in the maximum diameter where the elliptical region of interest incorporated the largest cross-sectional area of stone without including adjacent soft tissue. In our study, we determined MAV by measuring the mean HU of defined regions of interest just smaller than the stone in magnified images without including adjacent soft tissue on each slice of the axial planes (Fig. 1) with a standard bone window (window width-1,120 and window level-300) as suggested in the study by Eisner et al. [9]. We believe that this is the most accurate method of determining MAV. The inconsistent methods used in the literature might also explain the differing results that have been reported, so far (Table 2). In our opinion, image magnification for MAV measurement is very important because accurate stone margins can be identified using only adequately magnified images (Fig. 1). Thus, inclusion of adjacent soft tissue into measurement can be avoided. In addition, we measured all available slices of stones in axial planes to calculate MAV, which might prevent assumption of too high or low MAVs, as stones often consist of different components. The method of measuring MAV should be standardized to allow comparison of different datasets.

Concerning MAV, cut-off values between 750 and 1000 HU for renal calculi and between 750 and 900 HU in studies examining mixed ureteral and renal stones have been suggested as predictors of SWL failure (Table 2). However, separate examination of cut-off values for ureteral stones has only been performed in two studies: Pareek et al. [6] suggested 900 HU as the cut-off value in their study of 30 ureteral stones, and Ng et al. [7] defined a very different threshold of 593 HU as a potential predictor of treatment success in a study in 94 patients with upper ureteral stones. Our study failed to show an association between MAV and the disintegration of ureteral stones using SWL.

SSD has been shown to be a significant predictor of the outcome of SWL in different studies on renal stones. The findings for BMI, however, have been inconsistent as illustrated in Table 2.

Only two studies have analyzed SSD as a predictor of SWL treatment success in ureteral stones separately [5, 7] and both showed that SSD was a significant predictor. In this context, Ng et al. [7] suggested an SSD cut-off of 9.2 cm as a predictor for SWL failure, but they studied only upper ureteral stones.

In our study, SSD (90°) emerged as an even stronger predictor of treatment success or failure than mean SSD, with a cut-off value of 11.9 cm for SWL failure, which might be because we had patients in the almost straight supine or prone position for treatment with SLX-F2.

Results for BMI as a predictor of stone disintegration with SWL are also contradictory [5, 7]. In our study, BMI and patient weight were significant predictors of SWL outcome with cut-off values of 25.9 kg/m^2^ and 82.5 kg for SWL failure. Factors such as differences in body fat distribution between men and women, and age and race also have to be taken into consideration.

Possible limitations of our study are the retrospective design and assessment of disintegration outcome by KUB film and not by NCCT. Moreover, fragmentation rate instead of SFR was chosen to define treatment success or failure. We believed that this might represent the stone’s response to SWL better, because SFR is influenced by other factors that might interfere with stone passage (e.g., ureteral diameter). On the other hand, stone disintegration on KUB does not inevitably lead to a successfully completed treatment.

## Conclusions

The choice of treatment for ureteral stones should be based on stone location and size as considered in the AUA and EAU guidelines on urinary stone disease. Patient preference also has to be taken into consideration. In ambiguous cases, SSD and BMI – in contrast to MAV - can be easily used for additional guidance. In this way, patients with a high risk of disintegration failure could be educated more precisely, unnecessary exposure to shock waves and radiation could be avoided and medical costs could be reduced.

## Abbrevations

SSD: Skin-to-stone distance
MAV: Mean attenuation value
HU: Hounsfield Units
SWL: Shock wave lithotripsy
BMI: Body mass index
AUA: American Urological Association
EAU: European Association of Urology
SFR: Stone-free rate
URS: Ureterorenoscopy
NCCT: Noncontrast computed tomography
CT: Computed tomography
kV: Kilovolt
KUB: Kidney, ureter, and bladder film
ROC: Receiver operating characteristic
